# Understanding the relationship between Kano model’s customer satisfaction scores and self-stated requirements importance

**DOI:** 10.1186/s40064-016-1860-y

**Published:** 2016-02-27

**Authors:** Emmanuel O. C. Mkpojiogu, Nor Laily Hashim

**Affiliations:** Universiti Utara Malaysia, 06010 Sintok, Malaysia

**Keywords:** Kano model, Customer satisfaction coefficients/scores, Self-stated requirements importance, E-health awareness domain

## Abstract

Customer satisfaction is the result of product quality and viability. The place of the perceived satisfaction of users/customers for a software product cannot be neglected especially in today competitive market environment as it drives the loyalty of customers and promotes high profitability and return on investment. Therefore understanding the importance of requirements as it is associated with the satisfaction of users/customers when their requirements are met is worth the pain considering. It is necessary to know the relationship between customer satisfactions when their requirements are met (or their dissatisfaction when their requirements are unmet) and the importance of such requirement. So many works have been carried out on customer satisfaction in connection with the importance of requirements but the relationship between customer satisfaction scores (coefficients) of the Kano model and users/customers self-stated requirements importance have not been sufficiently explored. In this study, an attempt is made to unravel the underlying relationship existing between Kano model’s customer satisfaction indexes and users/customers self reported requirements importance. The results of the study indicate some interesting associations between these considered variables. These bivariate associations reveal that customer satisfaction index (SI), and average satisfaction coefficient (ASC) and customer dissatisfaction index (DI) and average satisfaction coefficient (ASC) are highly correlated (r = 96 %) and thus ASC can be used in place of either SI or DI in representing customer satisfaction scores. Also, these Kano model’s customer satisfaction variables (SI, DI, and ASC) are each associated with self-stated requirements importance (IMP). Further analysis indicates that the value customers or users place on requirements that are met or on features that are incorporated into a product influences the level of satisfaction such customers derive from the product. The worth of a product feature is indicated by the perceived satisfaction customers get from the inclusion of such feature in the product design and development. The satisfaction users/customers derive when a requirement is fulfilled or when a feature is placed in the product (SI or ASC) is strongly influenced by the value the users/customers place on such requirements/features when met (IMP). However, the dissatisfaction users/customers received when a requirement is not met or when a feature is not incorporated into the product (DI), even though related to self-stated requirements importance (IMP), does not have a strong effect on the importance/worth (IMP) of that given requirement/feature as perceived by the users or customers. Therefore, since customer satisfaction is proportionally related to the perceived requirements importance (worth), it is then necessary to give adequate attention to user/customer satisfying requirements (features) from elicitation to design and to the final implementation of the design. Incorporating user or customer satisfying requirements in product design is of great worth or value to the future users or customers of the product.

## Background

The place of users or customers’ satisfaction in software products development and the influence this holds in the quality of such products cannot be over emphasized. The import of this assertion implies that early investigation is needed to ascertain the requirements or features that will delight users and customers. This will avoid the risks associated with a late discovery of requirements or features that satisfy users or customers. It is important to find out the extent of users or customers satisfaction or dissatisfaction for product features before even the product is designed, developed and brought to the market to minimize or avoid risks like unnecessary rework or redesign, late product delivery, extra cost in terms of time, effort, personnel and finance, etc. Users or customers are great determinants of software product quality (Zhu et al. 2010). Products with features that satisfy and delight them are perceived as been of quality by them (Hussain et al. 2015). So, in the light of this, investigating the satisfaction that users or customers derive from software products before the product is even designed is not out of place as it saves cost and enables prompt delivery of user satisfying products to clients (Hussain et al. 2015). It is interesting to note that product quality is determined by customer satisfaction (Chen et al. 2013). Thus, issues on user or customer satisfaction are worth considering.

Knowing the extent of user or customers satisfaction is not enough, it is needful to also know the importance of (that is, the value the users or customers place on) the product requirements or features from the point of view of the user-customer stakeholder. This information provides a double barreled boost for the designs that succinctly delight users or customers and that also enhances the perceived quality of such products. Software quality, anchored on customer satisfaction, is crucial for the viability of software products and the survivability and sustainability of the software market (Chaudha et al. 2011); however, only a few studies examine critical issues of quality management from the customer or user’s perceptive (Issac et al. 2006). To get insight concerning what customers expect from a product, customers’ view point on the quality of such product is essential (Issac et al. 2006). This quality is captured from the extent of how satisfied or delighted users or customers are about a product (Zhu et al. 2010). Customers have specific expectations concerning the quality of the product they receive, particularly with regard to the other products that the given product is in competition with. This makes quality improvements necessary (Capell 2003). Software companies stand to gain when their customers are satisfied and delighted, but losses when their customers are dissatisfied as they will lose their patronage and loyalty (Rust and Oliver 2000). Studies show that product quality, customer satisfaction and customer loyalty are related (Olsen 2002).

In the following sub-sections, some basic concepts/issues are introduced and discussed in line with literature. These concepts/issues include: The Kano model, customer satisfaction, Kano model satisfaction coefficient and self-stated importance.

### Kano model

Kano model, developed by Professor Noriaki Kano, identifies and categorizes customer requirements or attributes as must-be, one-dimensional, attractive, indifferent and reverse requirements (or features). An understanding of these product quality attributes is beneficial to improving the quality of products and in the developments of products (Zhu et al. 2010). For example, Lee et al. (2015) discovered that Web portal preferences were driven by attractive, must-be, and one-dimensional qualities and Hussain et al. (2015) found that the requirements for an e-Ebola awareness Web portal are of attractive and one-dimensional types. This knowledge serves as a precursor to the design and development of quality products (Hussain et al. 2015). Requirements engineers need feedback from users on planned system features (Proynova and Paech 2013). These feedbacks are captured in a Kano survey (Hussain et al. 2015). The feedbacks are obtained before users have experience with the software product (Proynova and Paech 2013). Many researchers have applied Kano model to identify customer needs and how product attributes or features affects customer satisfaction (Gupta and Srivastava 2011). Kano model helps in identifying and distinguishing product requirements or features that have great influence on the satisfaction of customers. Classifying product requirements into must-be, attractive, one-dimensional, indifferent and reverse can be employed to focus on priorities for product development; for instance, it is not very useful to invest in improving must-be requirements that are already at the level of satisfaction, however, it is better to improve attractive and/or one-dimensional requirements, since they have a greater influence or impact on the perceived product quality and resultantly on the customers’ satisfaction level (Gupta and Srivastava 2011). However, Kano model’s classification of requirements is qualitative in nature and has little or no use in quantitative evaluation (Hussain et al. 2015).

Software engineering is a user-centric process that is potentially error prone. This error challenge can be minimized if adequate precaution is taken. Engaging the users in requirements elicitation is not sufficient without capturing, not only their voice but also their mind. Understanding how the end-users feel about the requirements and features of an intended system and modelling their level of satisfaction, is a necessary pre-requisite to software quality as unnecessary features that do not meet the real needs of the requirements of potential users are eliminated through this process. A model that does this well is the Kano Model, a two dimensional model. This approach as earlier mentioned, was proposed by Noriaki Kano. He based the model on Fredrick Hertzberg Two Factor Theory (Li-Li et al. 2011), and classifies user requirements into several categories with varying levels of impact on users satisfaction based on future fulfilment or non-fulfilment of the requirements. It is assumed that system functionalities are directly proportional to the users’ satisfaction (Lubinski and Oppitz 2012). Kano Model distinguishes what requirements or features bring satisfaction and what requirements have little association with satisfaction. It helps in identifying features and requirements of a proposed product that are most stimulating to users/customers (Nascimento et al. 2012; Li-Li et al. 2011; Miyuan et al. 2011; Lubinski and Oppitz 2012; Bi and Wang 2013).

Kano model plays a great role in understanding which software product can be used to attract a high degree of customer satisfaction and the product features that have a more than proportional influence on satisfaction as well as attributes that are an absolute must in the eyes of users or customers (Matzler et al. 1996). The model stipulates five key categories of requirements or product attributes. These requirement types define the perceived quality of proposed products. The Kano model requirements categories are: Must-be requirements: These set of requirements are the basic criteria of a product or the basic needs/expectations of the potential customers/users. They are the basic features customers/users expect from a software product. They are threshold requirements. The user or customer will be extremely dissatisfied if these requirements are not met or incorporated into the product design. However, customers take such requirements for granted and their fulfillment will not increase their satisfaction. Meeting must-be requirements only lead to a start of not being dissatisfied. Must-be requirements are regarded as pre-requisites by customers, thus, they take them for granted and therefore do not explicitly ask for them. This notwithstanding, must-be requirements are a decisive competitive factor. If they are not met, the user or customer will not be interested at all in the product (Hussain et al. 2015; Matzler et al. 1996).

One-dimensional requirements: These requirements are satisfiers. They are linear requirements. With respect to these requirements, customer satisfaction is proportional to the level of requirements or feature fulfillment. The more the fulfillment, the more the customer is satisfied and vice versa. This set of requirements is usually explicitly demanded for by users and customers (Hussain et al. 2015; Matzler et al. 1996). Attractive requirements: Attractive requirements are the product criteria that have the greatest impact on how satisfied a user or customer will be with a particular product. They are delighters or excitement requirements. This kind of requirements are neither explicitly expressed nor expected by users or customers. So, meeting these requirements leads to a more than proportional satisfaction. However, if these requirements are not fulfilled, there will be a feeling of dissatisfaction by the user or customer (Hussain et al. 2015; Matzler et al. 1996). Indifference requirement: This is a no preference requirement, which implies that the user/customer is indifferent to the requirement/feature. He or she does not care if the feature is present or not. The users do not actually care about this feature. This feature is neither good nor bad and they do not result in either the satisfaction or dissatisfaction of users/customers. Customers are not concerned with this requirement whether it is present or absent. Reverse requirement: Is an inverse requirement (that is, can be either way), here, the user/customer expectation about the feature is in a reverse order. The users prefer that the requirement would not be taken into account. These are the requirements/features that users’ do not expect. The more these features are met, the more dissatisfied users and customers will be. The requirements with a high degree of achievement result in dissatisfaction as all users are not the same/alike. With this requirement, customer’s/user’s satisfaction will be decreased. This requirement refers to a high degree of achievement that causes dissatisfaction. It always results in dissatisfaction (Hussain et al. 2015; Matzler et al. 1996).

As important and revealing as the Kano model is, it only shows the extent of user or customer satisfaction or dissatisfaction when requirements or features are met or unmet but do not show the importance in terms of the value the user or customer attach to the product requirements or features. Satisfaction and importance are not the same. While satisfaction deals with the performance of the requirements, importance (in the context of this study) has to do with the expected perceived value the requirements. This study examines the relationship between customer satisfaction (particularly, the Kano model customer satisfaction coefficient/scores) and requirements importance (particularly, users self-stated requirement importance).

Further to this, due to the qualitative nature of the Kano model and limitation of not being effective in the quantitative evaluation of customer satisfaction (Hussain et al. 2015), Berger et al. as cited in (Zhu et al. 2010) extended it for the computation of customer satisfaction coefficients. Customer satisfaction coefficient consists of two elements: (i) Satisfaction Index (SI) which explains the extent of satisfaction users or customers will derive from a product if the product requirements are met or implemented in the design of the product and (ii) Dissatisfaction Index (DI) that describes the degree of dissatisfaction that users or customers will get when the product requirements or features are not met or implemented in the product. SI and DI are both requirements performance indexes that describe the extent of a requirements performance. In addition, Park et al. (2012), summed the two indexes (that is SI and DI) to obtain Average Satisfaction Coefficient (ASC) that defines the degree of users or customers satisfaction of a product requirement or feature and that determines the importance value of such quality attributes (this coefficient, also defines the performance level of a software product) (Park et al. 2012). Some attempts have been made in finding the relationship between satisfaction and requirements importance in the literature, for instance (Zhu et al. 2010; Yang 2005). However, from literature so far searched, no work has been done on empirically finding out the relationship between customer satisfaction coefficient scores of the Kano model and self reported/stated importance of the requirements or features of proposed software products. In this regard, this study attempts to investigate such relationship and provide the implication associated with it especially in the e-health awareness domain.

A special Kano questionnaire is used to capture requirements. For each requirement/feature, a functional (positive) and a dysfunctional (negative) question are asked with five distinct possible answers/options for each. Through the responses to this questionnaire, there is the possibility of identifying the perceived level of user satisfaction for a given requirement (Nascimento et al. 2012). A Kano matrix is derived from the categorization of each feature/requirement depending on the combination of the answers to the functional and dysfunctional questions. This various categories stand also for the prioritization of each requirement (Nascimento et al. 2012). The Kano questionnaire probes both the customers’ voice and the customers’ mind; it explores the potential users’/customers’ psychology. Its use helps to filter out those requirements that will not satisfy the users’ needs or that have no value to the users, thus, enhancing the quality of the requirements; it provides a way of optimally deciding on which feature/requirement to retain and which to discard in the software development process. Kano model helps to extract from users what they really expect from a product. Using this model, proposed features that most stimulate users are captured; hence, enabling the building of a unique and lean product that contains the necessary features that pleases and at the same time amazes the users (Nascimento et al. 2012; Li-Li et al. 2011; Miyuan et al. 2011). Based on the Kano model, a customer satisfaction coefficient is calculated to obtain the percentage of users/customers who get satisfied with given requirements and those that don’t. The coefficient is used to assess the impact of the requirements/features on the satisfaction of the potential users of the given system (Li-Li et al. 2011; Miyuan et al. 2011; Lubinski and Oppitz 2012; Bi and Wang 2013).

### User/customer satisfaction

User or customer satisfaction is hinged on meeting the needs and expectation and or even exceeding the expectations of user or customers for a given software product. Where such criteria are met, there is delight on the part of users or customers and such delight propels them to patronize the product and be loyal users or customers to the product. This consequently enhances the return on investment and profitability of the software company. Oliver (1977) expectancy disconfirmation theory conceptualized satisfaction as a measure of interaction between a customer’s pre-purchase expectation and post-purchase evaluation, where satisfaction results from perceived outcomes exceeding expectation. Positive disconfirmation occurs when product performance is better than expected, while negative disconfirmation occurs when product performance is less than expected (Oliver 1977) According to Oliver (1977), satisfaction is caused by positive disconfirmation of customer expectations while dissatisfaction is caused by negative disconfirmation of customer expectations (Oliver 1977). Parasuraman et al. (1985) used the comparison between the customers’ perceived value with their expectation based on the five dimensions: reliability, tangibility, empathy, responsiveness and assurance to assess the product/service quality (Parasuraman et al. 1985). Customers are likely to see products as a collection of attributes that vary in their level of contribution to the satisfaction of the user or customer. Taplin (2012) stressed that satisfaction is determined by performance on a variety of specific attributes or components. Hence, increasing the performance of attributes with higher performance will more likely increase overall satisfaction (Taplin 2012). Satisfaction promotes product loyalty and goodwill and paves way for increasing improvement in software product quality (Hussain et al. 2015). In addition, satisfaction has also been measured using gap between performance and expectations [Brady et al.; Wang et al. as cited in Taplin (2012)]. However, this satisfaction measurement has been questioned [Tian-Cole et al.; Spreng et al., as cited in Taplin (2012)]. Kano model captures product satisfaction from a different angle. It captures how customers feels if a proposed feature or attributes is in a product and also how they feel if such feature or attributes will not be present in the product. It obtains the customers’ satisfaction apriori, that is, even before they experience the product (Zhu et al. 2010; Matzler et al. 1996).

Matzler et al. (1996) argued that customer satisfaction in a powerful indicator for the future of any business. They opined that investigating and striving for customer satisfaction implies understanding and anticipating what customers want from a product in the future. Delighting customers with software products that engender very positive response and surprise in them have a good pay back (Matzler et al. 1996). Matzler et al. (1996) further reiterated that satisfied users and customers are loyal users and customers and that scenario ensures a lasting cash-flow for the business in the long run. They also stressed that satisfied customers are less sensitive to price and more likely to buy more and are more inclined to spend more on tested and tried products. Product satisfaction promotes positive quality image and lowers the cost of attracting new customers. Satisfaction influences repeat purchase customer behavior and loyalty. High level of user or customer loyalty reduces the cost of transaction for existing customers (Matzler et al. 1996).

Software quality as postulated by the IEEE (1991) is defined as:

- “The degree to which a system, component, or process meets specified requirements”
- “The degree to which a system, component, or process meets customer or user needs or expectation”

Each of the definitions above indicates a different view and concept of software quality (Galin 2004). From the second part of the definition, it can be deduced that customer satisfaction depends on the quality of the software product delivered to them. Quality is expressed as the meeting of users’/customers’ needs/expectations and their resultant derived satisfaction from the software product. Quality is stressed and hinged on customer satisfaction and conforming to the expectations of users and customers.

### Kano model’s coefficient of customer satisfaction

As pointed out earlier, the Kano model is limited because it evaluates customer satisfaction qualitatively. This limitation prevents a quantitative assessment of the extent of customer satisfaction with regard to if requirements are met or unmet. A number of improvements have been done on the original Kano model to incorporate quantitative evaluation of customer satisfaction. See Hussain et al. (2015) for a review of some of them. For the purpose of this paper, the works of Berger et al. and Park et al. will be examined.

Berger et al. (1993) improved on the Kano model by developing the customer satisfaction coefficient (CS) for a given requirement. The CS explains whether satisfaction can be increased by meeting the requirements of a product or whether meeting the requirements of the product merely hinders the customers from being satisfied (Berger et al. 1993). The CS provides the average impact of a product requirement on the satisfaction of all users or customers. It gives an indication of how strongly a product feature may impact on user or customer satisfaction or on the other hand, how strongly the non-fulfillment of a product requirement or feature may influence user or customer dissatisfaction. The former is called the satisfaction index (SI) while the latter is called the dissatisfaction index (DI) (Matzler et al. 1996). SI is positive CS-coefficient while DI is negative CS-coefficient. The following are the computation formula for SI (extent of satisfaction) and DI (extent of dissatisfaction) as defined by (Berger et al. 1993):

1 $$ {\text{SI}} = {{\left( {{\text{A}} + {\text{O}}} \right)} \mathord{\left/ {\vphantom {{\left( {{\text{A}} + {\text{O}}} \right)} {\left( {{\text{A}} + {\text{O}} + {\text{M}} + {\text{I}}} \right)}}} \right. \kern-0pt} {\left( {{\text{A}} + {\text{O}} + {\text{M}} + {\text{I}}} \right)}} $$

2 $$ {\text{DI}} = \, {{\left( { - 1} \right)\left( {{\text{O}} + {\text{M}}} \right)} \mathord{\left/ {\vphantom {{\left( { - 1} \right)\left( {{\text{O}} + {\text{M}}} \right)} {\left( {{\text{A}} + {\text{O}} + {\text{M}} + {\text{I}}} \right)}}} \right. \kern-0pt} {\left( {{\text{A}} + {\text{O}} + {\text{M}} + {\text{I}}} \right)}} $$

where SI is the extent of satisfaction (satisfaction index), DI is the extent of dissatisfaction (dissatisfaction index), A is attractive requirement, O is one-dimensional requirement, M is must-be requirement and I is indifferent requirement. The minus sign in the DI equation (Eq. 2) is to place emphasis on the negative influence on user/customer satisfaction that will result if the requirement is not met or if the feature is not incorporated into the design of the product.

The positive CS-coefficient (SI) ranges from zero to one. The nearer the value is to one, the greater the impact of the meeting of the requirement on customer satisfaction, but the closer the value is to zero, lesser the influence. Also, the negative CS-coefficient (DI) ranges from zero to minus one. The closer the value is to minus one, the higher the influence of the non-fulfilling of requirements on customer dissatisfaction. The minus sign in front of Eq. (2) is for the purpose of putting emphasis on the negative effect of not meeting requirements on customer satisfaction. A zero score implies no influence on customer satisfaction whether or not the requirement is met or unmet (Matzler et al. 1996). Berger et al. (1993) did not examine the correlation between SI and DI. This study among others objectives, seeks to find out the degree of relationship that exists between SI and DI.

Park et al. (2012) in attempting to determine the performance of quality attributes developed another model that averages the sum of the absolute value of both the positive CS-coefficient (SI) and the negative CS-coefficient (DI) to obtain average satisfaction coefficient (ASC). They however did not indicate how the new index (ASC) correlates with SI and DI. In this study, the level of correlations of the three indexes (SI, DI, and ASC) is examined. The Park et al. (2012) equation is as follows:

3 $$ {\text{ASC}} = {{\left( {\left| {\text{SI}} \right| + \left| {\text{DI}} \right|} \right)} \mathord{\left/ {\vphantom {{\left( {\left| {\text{SI}} \right| + \left| {\text{DI}} \right|} \right)} 2}} \right. \kern-0pt} 2} $$

### Self-stated requirements importance

From literature, there are two types of requirements importance, namely: i) stated (reported) and ii) statistically inferred importance (derived importance). Self-stated importance is a self-reported importance. It is a direct method where requirements importance is captured directly from customers using a scale (Pezeshki et al. 2009). That is, the value of the requirement or feature as perceived by the user or customer that is stated by word of mouth. Grigoroudis and Spyridaki (2003), defines self-stated importance as “the straightforward customer preferences for the weight of a satisfaction criteria”. In the stated importance method, users or customers are asked to rate the importance of the requirement, attribute or feature on a Likert scales typically ranging from “not important at all” to “very important”. However, this approach has some weakness. Customers tend to assign higher importance to attributes or features that represent basic functionality (Tontini and Silveira 2007). Stated importance usually tends to have low discrimination and customers are likely to find everything important. This happens because users or customers normally reason about the importance of requirements or features in terms of how they result into socially acceptable outcomes or at least maintain status quo, Garver as cited in (Tontini and Silveira 2007). However this bias is resolved or at least minimized by the use of higher sample sizes and by use of trained and skilled interviewers. Martilla and James as cited in (Tontini and Silveira 2007) used self-stated importance and performance of attributes with a two-dimensional plot (Tontini and Silveira 2007). Self-stated importance can be measured using an easy method that uses questionnaire that is designed using 7-point Likert-type scale that ranges from 1-totally unimportant to 7- very important. This instrument is used to capture user/customer stated requirement importance (Matzler et al. 1996).

The statistically inferred importance is derived by statistical means, for example, beta values of a regression analysis, statistical correlations or structural equation modeling (Taplin 2012). This is an indirect method that is hinged on the relationship between performance of attributes and customer satisfaction (Pezeshki et al. 2009). Grigoroudis and Spyridaki (2003) states that derived importance is estimated by a regression-type quantitative technique using customer judgments for the performance of the criteria. With respect to the statistically inferred importance, customers are asked to rate both their satisfaction with the current performance of the different requirements or features or attributes and their overall satisfaction with the requirement, feature or attribute. Researchers have used several approaches in capturing this kind of importance, namely: multiple regressions, partial least squares with reflective or formative attribute specification, pair-wise estimation, and principal component regression, etc., Gustafson and Johnson as cited in (Tontini and Silveira 2007). Requirements or attributes with higher regression coefficients are deemed to be more important than others. Although this approach removes the tendency of getting all attributes important and also discriminates better the relative importance between them, Garver as cited in (Tontini and Silveira 2007), it is still deficient. There is usually the occurrence of multi co-linearity among independent variables, which tends to be problematic. Also, the relationship between the satisfaction with an attribute or feature and overall satisfaction do not always follow linearity (Tontini and Silveira 2007). In addition, customers tend to say they are “satisfied” when in reality they are not, but actually in a neutral state. This leads to bias in the data (Tontini and Silveira 2007). In this study, the self-stated importance approach is employed. The bias observed in this review was controlled by using a fair sample size and utilizing a trained and skilled interviewer.

### Measuring the relationship between user/customer satisfaction and importance

The importance that customers or users attach to software products is an important part of customer satisfaction (Cugnata and Salini 2014). In measuring the relationship between user or customer satisfaction and requirements’ importance, several methods are used. In the literature, most researchers employ the use of importance-performance analysis (IPA) method (Zhu et al. 2010; Tontini and Silveira 2007; Pezeshki et al. 2009). Yang (2003) used importance-satisfaction model. The IPA was originally introduced by Martilla and James as cited in (Tontini and Silveira 2007). The method allows a company to identify which attributes or features of its products should be improved so as to be more competitive in the market. Data from customer survey are used to build a matrix. In this matrix, importance is shown on the y-axis while attribute or feature performance is shown on the x-axis (Tontini and Silveira 2007). In the case of Yang (2003), satisfaction is shown on the y-axis while importance is shown on the x-axis, with the mean of each variable placed along the respective axis, and dividing the graph into four quadrants (areas) (area I, excellent area; area II, to be improved area; area III, surplus area; area IV, careless area) (Yang 2003). In the traditional IPA, the matrix is split into four quadrants. Attributes with high importance and high performance (satisfaction) are located in quadrant I. this represents a possible competitive advantage. Attribute with high importance but with low performance (satisfaction) are located in quadrant II. This set of attributes is to receive immediate attention. Also, attributes with low importance and low performance (satisfaction) are located in quadrant III. These requirements or features do not need additional effort on them. Lastly, attributes with low importance and high performance (satisfaction) are located in quadrant IV. Giving attention to these set of attributes and features would mean a company wasting resources that should have been channeled or better used in other places (Tontini and Silveira 2007). However, the drawback of this quadrant approach is that “a minor change” in the position of an attribute may lead to a drastic change in the attribute’s inferred priority, Eskildsen and Kristensen as cited in (Tontini and Silveira 2007). Models used in satisfaction analysis include: SERVQUAL model, importance-satisfaction model and importance-performance model (Setijono 2008; Chen et al. 2006).

Yang (2005) observed a deficiency in the traditional Kano’s model that prevents firms from precisely evaluating the influences of quality attributes. He opined that the weakness was a failure to account of the degree of importance accorded to certain quality elements by customers. He refined the Kano’s model, added the importance of quality attributes, and in so doing, the refined model divides Kano’s first four categories into eight categories, namely, highly attractive and less attractive, high value-added and low value-added, critical and necessary, potential and care-free. He concludes that based on the refined model, firms can obtain a more accurate understanding of the quality attributes from customers’ perspective and can thus make more precise quality decisions. Yang (2005) developed an importance-satisfaction (I-S) model and integrated his refined Kano’s model and the I-S model, thus enabling firms to gather more valuable information on quality decisions (Yang 2005). Slack as cited in (Tontini and Silveira 2007) proposed a different approach to analyze the IPA matrix. This is done by dividing the matrix into non-symmetric action zones. In this approach, the importance and the performance axes are inverted so as to be compatible with the traditional IPA.

A major drawback with IPA approach is that it leads to different conclusions depending on how an attribute’s importance is figured out. More so, it does not consider the non-linear correlation between the performance of the attributes and customer satisfaction. Hence, this leads to misleading improvement decisions and a hindrance to innovations. The Kano method however, identifies the non-linear correlation between performance (satisfaction) and attributes (requirements), but do not take into account the current level of requirements or attributes’ performance in its analysis.

In the present study, the interest is not on capturing the present level of requirements performance but rather to obtain a predictive measure (models) of the requirement performance (satisfaction) as a proxy for the perceived quality of a proposed product, using the perceived requirements importance as an explanatory variable and customer satisfaction as the dependent variable. Capturing this will enable planning and design of quality software products and to prevent the risks of unnecessary rework and its associated costs. So, in this study, the performance (satisfaction) of requirements is captured through the Kano method as it suits into the scope of the study, while importance (requirements value/worth) is captured using a single question perception questionnaire (self-stated requirements importance rating questionnaire). Both variables were elicited during the Kano survey.

The remaining part of this paper dwells on the following sections: Second section presents the methodology employed in this research; Third section describes the synopsis of how the empirical study was conducted; Fourth section covers results and discussion, this includes, socio-demographic information, correlation and regression analyses; and lastly, Fifth section provides the conclusion. This also includes summary, limitation of the study and future works.

## Methodology

In this paper, Kano Model was used for data collection and analysis. Berger et al. (1993) extension of Kano Model (Kano et al. 1984) was applied in determining the coefficient of customer satisfaction. A Kano questionnaire [based on the Kano model (Matzler et al. 1996)] was constructed and administered to fifty (50) respondents during the Kano survey and survey ethics were duly observed. The survey was designed to capture requirements for an E-Ebola Awareness System, an online e-Health awareness system. It is intended that this system will be built with quality in mind, right early from the elicitation of requirements. The requirement elicitation process takes the end-user into account such that the requirements gathered are does that satisfy and delight them. The Kano questionnaire design was adopted from (Matzler et al. 1996). The research participants were staff and students of Universiti Utara Malaysia, Malaysia. All participants are potential users of the proposed e-Ebola Awareness System (E-Easy) and all had pre-knowledge of the Ebola Virus Disease. With the sample size of fifty (50) the expected margin of error was 13 %. To remove inconsistency and bias, two questions were asked for each requirement elicited (following the Kano model); one of the questions was a functional (positive) question while the other was dysfunctional (negative). Each question (functional or dysfunctional), has a list of five options that respondents can select from. The options are: (1) I like it; (2) it must be; (3) I am neutral; (4) I can live with it; (5) I dislike it. The first question was asked to find out how the users/customers feel if the proposed feature is in place or requirement is met while the second question was to find out how they feel if the intended feature is not in place or requirement is not met. Each of the questions (whether functional or dysfunctional), has a list of five options, namely: (1) “I like it that way” (like), (2) “It must be that way” (must-be), (3) “I am neutral” (neutral), (4) “I can live with it that way” (live with), (5) “I dislike it that way” (dislike). After the survey, the result was tallied and totaled to show how the majority of users/customers expressed their requirements, and this was categorized into M “Must-Be”, O “One-Dimensional”, A “Attractive”, I “Indifferent”, R “Reverse” and Q “Questionable” categories. Concerning the “Questionable” category, the user has no opinion on the requirement. The answer to this question in the questionnaire by the potential user does not really make sense. They represent contradictions or misunderstandings in the requirements and indicate incorrectly phrased requirements, misunderstanding of requirements, or an incorrect response. The highest tally/count among the totals of each of these categories for a given requirement is picked as the category for the requirement. The rule for evaluation is: “M > O > A > I”. This rule guides decisions when making decisions on which feature/requirement has more influence on the perceived quality of the proposed software product (especially when their values are the same) (Li-Li et al. 2011; Supply Chain Opz 2015).

During the questionnaire administration, a screening question was asked to screen out those who are not eligible to respond to the questionnaire. The screening question was: “Have you heard of Ebola in the past?” Only respondents that responded “Yes” were eligible to respond to the Kano questions. After the survey, the responses were collated and analyzed using a semi-automated Kano Analysis Excel tool. Further analysis was done with SPSS version 17 package. In addition, the requirements were categorized following Kano’s approach (Kano et al. 1984) (see Table 1) and the Coefficient of User/Customer Satisfaction was computed using Berger et al. (1993) method (see Table 2). Five requirements/features were elicited in the Kano survey [for details on the elicited requirements, see Hussain et al. (2015)]. Also, a self-stated importance was elicited from participants using a 7-point Likert-type importance rating scale that ranges from totally unimportant to very important using the design methodology in Matzler et al. (1996) (see Table 3).

**Table 1:** Kano requirements categorization table (Hussain et al. 2015)

Requirements	M	O	A	I	R	Q	Total	Category
R1	11	14	16	06	03	–	50	A
R2	5	15	13	14	03	–	50	O
R3	07	22	11	07	02	01	50	O
R4	07	22	12	06	03	–	50	O
R5	04	18	06	18	04	–	50	O

Requirements	M %	O %	A %	I %	R %	Q %	Total %	Category
R1	22	28	32	12	06	–	100	A
R2	10	30	26	28	06	–	100	O
R3	14	44	22	14	04	02	100	O
R4	14	44	24	12	06	–	100	O
R5	08	36	12	36	08	–	100	O

*A* (attractive), *O* (one-dimensional), *M* (must-be); *I* (indifference), *R* (reverse); *Q* (questionable), *R1–R5* (requirements)

**Table 2:** Table of customer satisfaction coefficients (Hussain et al. 2015)

Customer Satisfaction (CS) Coefficients		
Requirements	SI	DI
R1	.64	−.53
R2	.60	−.43
R3	.70	−.62
R4	.72	−.62
R5	.52	−.48

*R1–R5* (requirements)

**Table 3:** Respondents’ self-stated importance (Hussain et al. 2015)

Self-stated importance rating (IMP)			
Requirements	Mean	Std	Rank
R3	5.74	1.34	1
R4	5.32	1.71	2
R1	5.32	1.48	3
R2	5.28	1.47	4
R5	5.04	1.59	5

*R1–R5* (requirements)

The entire survey instrument was checked and examined for reliability using Cronbach Alpha and the result was 0.79, indicating a good internal consistency of the questionnaire items. The Cronbach Alpha coefficient is usually used in computing the reliability of a survey instrument. A Cronbach Alpha values of 0.7 and above are accepted as acceptable reliability coefficients (Nunnaly 1973).

The Berger et al. improvement of the Kano method was employed to compute the coefficient of customer satisfaction (CS) using Eqs. (1) and (2). Equation (1) calculates the customer satisfaction index (SI) while Eq. (2) calculates the customer dissatisfaction index (DI). Park et al. model was used to compute the average satisfaction coefficient (ASC) utilizing Eq. (3).

After obtaining the SI, DI and ASC, a Pearson (r) correlation analysis was carried out to determine the relationship among the indexes/scores. In addition, the degree of the relationships was also captured and examined. In addition, from the same survey as stated earlier, a self-stated importance (IMP) rating for requirements was collected. This data was used along with the data on customer satisfaction to compute the correlation between customer satisfaction scores and self-stated requirements importance. Furthermore, a simple regression analysis was also conducted to ascertain the level of impact or influence that self-stated requirements importance (IMP) has on the satisfaction of users or customers if requirements are met and when they are not met. The self-stated requirements importance was used as an independent variable while SI, DI and ASC were used as dependent variables. SI, DI and ASC represent customer satisfaction, while IMP represents perceived requirements importance. Also, the equation was switched and SI, DI, and ASC were individually regressed on IMP to observe their effect on IMP (in order to obtain a balanced picture of the behavior of the observed variables).

## Empirical study

In carrying out this study, fifty participants were sampled purposively for the questionnaire administration. As afore stated in the methodology, a mix of both students and staff of Universiti Utara Malaysia from different demographic backgrounds, were used as study respondents to elicit requirements for a proposed e-ebola awareness system. These participants were used because they are all potential users/customers of the proposed software product. All participants were asked a screening question to ascertain whether they have any knowledge on ebola. Only participants who claim they have such knowledge were conscripted into the survey. The survey instruments used were in two parts: Kano questionnaire and self-stated requirements importance questionnaire. These two sections of the study instrument were administered to each participant. Next, Kano quality attributes were obtained through the analysis and categorization of the Kano questionnaire. The customer satisfaction indexes were computed from the Kano categorization statistics; while on the other hand, the perceived requirements importance ratings were captured and computed from the requirements importance question. Sequel to this, the Kano model customer satisfaction scores (indexes) were correlated with the perceived (self-stated) requirements importance ratings to verify the underlying association existing between the Kano model customer satisfaction scores and the self-stated requirements importance ratings. Furthermore, each of the Kano model customer satisfaction scores (SI, DI, and ASC) were regressed on self-stated requirements importance (IMP) to assess the effect of the self-stated importance ratings on the customer satisfaction scores.

## Results and discussion

This section presents the results of analysis and discussion. Here, the socio-demographic characteristics of respondents are presented and discussed as well as the results of the correlation and regression analysis. The survey data used in the correlation and regression analyses are summarized in Tables 1, 2, and 3.

### Demographic characteristics

The outcome of the survey reveals that out of the 50 respondents, 58 % were male and 42 % were female. Their age distribution is as follows: below 20 (4 %), 20–29 (64 %), 30–39 (26 %), 40–49 (2 %), above 49 (4 %). Their marital status are inter alia: single (70 %), married (30 %). The respondents’ highest educational qualification is distributed as follows: Secondary School Certificate (22 %), Diploma (6 %), Bachelor (50 %), Masters (18 %), Doctorate (4 %); and the spread of their monthly income in Malaysian Ringgit is as follows: Below 3000 (86 %), 3000–7000 (12 %), 7001–10000 (2 %).

These demographic statistics shows the spread and distribution of participants and the coverage of their background. With regard to their gender, both males and females were well represented. Their age distribution indicates that most of the participants were within the ages of 20–29 (64 %) and 30–39 (26 %). More so, a larger percentage of respondents were singles (70 %), the married were only 30 %. In addition, the proportion of the highest educational qualification of participants was more for Bachelor degree holders (50 %), followed by secondary school certificate holders (22 %). With respect to monthly income, majority of the respondents claimed that their monthly income was below RM3000 (86 %), this category is followed by those in the RM3000-RM7000 category of monthly income (12 %). The peculiarity and characteristics of these demographics is due to the setting of the survey.

On a general note, the distribution of participants was comfortably suitable for the study of this nature since they cover a considerable spectrum or segment of potential users of the proposed software product (with regard to gender, marital status, education etc.). The spread of the countries of origin of participants is as follows: Nigeria (18 %), Yemen (10 %), Somalia (6 %), Iraq (10 %), China (4 %), Malaysia (32 %), and Oman (6 %). Others consist 14 %, which comprise: Pakistan, Zimbabwe, Libya, Jordan, Algeria, Indonesia and Thailand, each with 2 % proportion. The participants’ countries of origin were mostly from Africa (30 %), Asia (42 %) and the Middle East (28 %), cutting across the continents of Africa (30 %) and Asia (70 %).

These demographics are essentially significant because the participants came from areas where information concerning the Ebola Virus Disease is likely to be sought for. It is interesting to know that past occurrence of the Ebola Virus Disease was concentrated in one the continent captured in the survey. In addition, the impact of a recent Ebola crises in some parts of the world was felt virtually everywhere in almost every continent, so, the participants must have responded to the Kano survey with a good knowledge of the effect of the Ebola Epidemic.

In this analysis, Pearson (r) correlation analysis was computed first, followed by the simple regression analysis. Each analysis was followed or accompanied by a follow-up discussion.

### Correlation analysis

The rational for the correlation analysis is to assess the relationship that exists among the Kano model’s customer satisfaction variables (SI, DI and ASC) and between these variables and the self-stated requirements importance variable (IMP).

Table 4 provides the two tail Pearson, r, correlation analysis for SI, DI, ASC and IMP variables. SI and ASC (r = 0.96), SI and IMP (r = 0.91), and ASC and IMP (r = 0.90) are significantly related, p < 0.05. Also, DI and ASC (r = 0.96) are significantly related at p < 0.01. However, SI and DI and DI and IMP are not significantly associated in a two tail relationship. Also Table 5 corroborated with Table 4 and further explains the relationship between SI, DI, ASC, and IMP in a one-tail Pearson (r) relationship. SI and IMP (r = 0.91), and ASC and IMP (r = 0.90) are significantly correlated, p < 0.05. SI and ASC (r = 0.96) and DI and ASC (r = 0.96) are also significantly correlated, p < 0.01. However, unlike the result in Table 4 (two-tail relationship), there is a significant one-tail relationship between SI and DI (r = 0.84), and DI and IMP (r = 0.81) at p < 0.05. As can be observed, there is a very high correlation between SI and ASC and DI and ASC (both have r = 0.96). This implies that ASC is similar to SI and DI and can be used interchangeably (that is, ASC can be used in place of either SI or DI and vice versa, to represent customer satisfaction). Also, IMP correlated well with all other variables (SI, DI, and ASC) in one-tail relationship. It does the same in two-tail relationship, except in the case of the correlation of DI and IMP where it is not significant. This means that an increase in the dissatisfaction users or customers have due to requirements not met, is associated to the increase in value they place on the requirement or feature. However, a decrease in such dissatisfaction does not lead to increase in the importance (value) of the requirements or feature from the view point of the users or customers (so the relationship between DI and IMP is only one directional.) But the correlation between SI and IMP and ASC and IMP are two directional. Also SI and DI are only related in one direction, that is, the increase in the satisfaction users or customers will derive from requirements or features that are met is proportional to the dissatisfaction that they will receive if such requirements or features are not fulfilled. But the reverse is not the case.

**Table 4:** A two-tail Peason (r) correlation analysis for SI, DI, ASC and IMP

Variables	SI	DI	ASC	IMP
SI	1			
DI	.836 (.078)	1		
ASC	.956 (.011)*	.960 (.010)**	1	
IMP	.914 (.030)*	.808 (.098)	.897 (.039)*	1

p values in (), p < 0.01 level**; p < 0.05 level*

**Table 5:** A one-tail Pearson (r) correlation analysis for SI, DI, ASC and IMP

Variables	SI	DI	ASC	IMP
SI	1			
DI	.836 (.039)*	1		
ASC	.956 (.006)**	.960 (.005)**	1	
IMP	.914 (.015)*	.808 (.049)*	.897 (.019)*	1

p values in (), p < 0.01 level**; p < 0.05 level*

### Regression analysis

The basis of the models used in the paper is to assess the level of impact of self-stated requirements importance (IMP) on customer satisfaction [represented by the three Kano model’s customer satisfaction variables (SI, DI and ASC)]. It is also to find out how these Kano model’s variables relate to each other and to IMP. Also, the influence three Kano customer satisfaction variables on self-stated requirements importance were also assessed so as to double check and have a bi-directional picture of the behavior of the variables in the model.

Tables 6 and 7 explain the model: SI_i_ = α + β(IMP_i_) + e_i_. The model fits well the data, F(1,3) = 15.125, p < 0.05. IMP has a significant effect on SI, t(3) = 3.889, p < 0.05. However, the constant, α, is not significant. This result implies that a unit change (increase) in the worth of the requirement (feature) as perceived by the user or customer is associated with an average of 0.279 units of satisfaction if the requirement is met or if the feature is included in the product design. The value that potential users/customers of a software product place on a given requirement or feature goes a long way in determining how satisfied they will be with the product. So, it will be very needful to elicit and find out what these requirements or features are, and measure their level of worth, even before the design of the product, to better capture the satisfaction of the intended or potential users/customers of the product. In addition, the user or customer will not be satisfied if requirement is not fulfilled or if the feature is not incorporated into the product (as seen from the negative α coefficient). This underscores the need to met and fulfill the requirements that are discovered to be of importance to the users/customers in the product design and development as failure to do so will result in the dissatisfaction of users/customers and will in turn affect their perception of the product quality. In Tables 8 and 9, the model IMP_i_ = α + β(SI_i_) + e_i_ was fitted significantly to the data, F(1,3) = 15.123, p < 0.05. SI has a significant influence on IMP, t(3) = 3.889, p < 0.05. This implies that a change (increase) in satisfaction if the requirement is met or if the feature is present in the software product will lead to an increase in the perceived value of the feature (by 2.994 units) in the eyes of the user/customer. That is, when users/customers are satisfied with requirements that are incorporated into a product, it speaks clearly about how such users/customers treasure the said requirements. This means that user satisfaction impacts on the importance of requirements as perceived by users/customers. There is a direct proportionality between user/customer satisfaction and the perceived importance of requirements. However, if no satisfaction is derived from the meeting of the requirement, there will still be significant increase in the importance (worth) of the feature due to some other unexplained factors other than satisfaction, that are not included in the model.

**Table 6:** ANOVA table for SI_i_ = α + β(IMP_i_) + e_i_; R = .914; R^2^ = .834; Adjusted R^2^ = .779; Std Error = .038

Model	Sum of square	df	Mean square	F	p value
Regression	.022	1	.022	15.125	.030
Residual	.004	3	.001		
Total	.026	4

**Table 7:** Regression table for SI_i_ = α + β(IMP_i_) + e_i_

Model	β (unstandadized)	Std error (of β)	β (standardized)	t	p value
IMP	.279	.072	.914	3.889	.030
α	−.864	.386		−2.238	.111

**Table 8:** ANOVA table for IMP_i_ = α + β(SI_i_) + e_i_; R = .914; R^2^ = .834; Adjusted R^2^ = .779; Std Error = .124

Model	Sum of square	df	Mean square	F	p value
Regression	.232	1	.232	15.125	.030
Residual	.046	3	.015		
Total	.278	4

**Table 9:** Regression table for IMP_i_ = α + β(SI_i_) + e_i_

Model	β (unstandadized)	Std error (of β)	β (standardized)	t	p value
SI	2.994	.770	.914	3.889	.030
α	3.476	.493		7.055	.006

Furthermore, Tables 10 and 11 explains the model: DI_i_ = α + β(IMP_i_) + e_i_. The fitting of the model is not significant even though IMP accounts for 54 % of the variability in DI (adjusted R^2^ = 0.537). IMP does not have any significant influence on DI. The correlation of DI and IMP (r = β = 0.81) is not significant. In Tables 12 and 13, IMP_i_ = α + β(DI_i_) + e_i_ model does not significantly fit well into the data, even though IMP account for 54 % of the variability in DI (adjusted R^2^ = 0.537). IMP does not have any significant impact on DI. However, the constant, α, is very significant, t(3) = 7.00, p < 0.01. The association of IMP and DI (r = β = 0.81) is not significant. This result shows that user/customers dissatisfaction as a result of requirements not being met does not imply that the requirement/feature is not important as seen from the significant α coefficient, which indicates that some other factors (other than dissatisfaction) not captured in the model could be responsible for the worth of the requirement/feature.

**Table 10:** ANOVA table for DI_i_ = α + β(IMP_i_) + e_i_; R = .808; R^2^ = .653; Adjusted R^2^ = .537; Std Error = .057

Model	Sum of square	df	Mean square	F	p value
Regression	.019	1	.019	5.643	.098
Residual	.010	3	.003		
Total	.029	4

**Table 11:** Regression table for DI_i_ = α + β(IMP_i_) + e_i_

Model	β (unstandadized)	Std error (of β)	β (standardized)	t	p value
IMP	.259	.109	.808	2.376	.098
α	−.855	.586		−1.459	.241

**Table 12:** ANOVA table for IMP_i_ = α + β(DI_i_) + e_i_; R = .808; R^2^ = .653; Adjusted R^2^ = .537; Std Error = .179

Model	Sum of square	df	Mean square	F	P-value
Regression	.182	1	.182	5.643	.098
Residual	.097	3	.032		
Total	.278	4

**Table 13:** Regression table for IMP_i_ = α + β(DI_i_) + e_i_

Model	β (unstandadized)	Std error (of β)	β (standardized)	t	p value
IMP	2.525	1.063	.808	2.376	.098
α	4.027	.575		7.000	.006

Further still, Tables 14 and 15 provides an explanation for the ASC_i_ = α + β(IMP_i_) + e_i_ model. The data fits well into the model, F(1,3) = 12.373, p < 0.05. IMP accounts for 74 % of the variability in ASC. Also, IMP has a significant influence on ASC, t(3) = 3.518, p < 0.05. But the constant, α, is non-significant. This analysis shows that an increase in the worth (importance) of requirements/features leads to a corresponding increase in the average satisfaction users/customers will derive from a product if the particular requirements are met or the given features are put into the design of the product. The result also implies that the increase in the value of a requirement/feature as perceived by users/customers is proportional to the increase in the dissatisfaction they will receive if such requirement/feature is not implemented in the software product. Tables 16 and 17 explain the model: IMP_i_ = α + β(ASC_i_) + e_i_. The model fits well into the data, F(1,3) = 12.373, p < 0.05. ASC accounts for 74 % of the variability in IMP. Also, ASC has a significant effect on IMP, t(3) = 3.518, p < 0.05. In addition, the constant, α, is also very significant, t(3) = 7.211, p < 0.01. This result reveals that an increase in average satisfaction derived from a fulfilled requirement or the dissatisfaction caused by the non-fulfilment of the requirement will lead to proportional increase in the importance of the given requirement/feature as perceived by users/customers of the proposed product. This implies that, the satisfaction users/customers get when their needs and expectations are met or the dissatisfaction they have when the reverse is the case, determines or indicates how important the requirements/features (needs/expectations) are to them. This will necessitate ensuring that from elicitation to design and development phase of the product; such requirements are captured, ascertained and built into the product. Furthermore, the absence of such satisfaction/dissatisfaction, can also lead to an increase in the importance of the requirement/feature due to some other factors not captured in the model (as seen from the significant constant, α).

**Table 14:** ANOVA table for ASC_i_ = α + β(IMP_i_) + e_i_; R = .897; R^2^ = .805; Adjusted R^2^ = .740; Std Error = .0403

Model	Sum of square	df	Mean square	F	p value
Regression	.020	1	.020	12.373	.039
Residual	.005	3	.002		
Total	.025	4

**Table 15:** Regression table for ASC_i_ = α + β(IMP_i_) + e_i_

Model	β (unstandadized)	Std error (of β)	β (standardized)	t	p value
IMP	.269	.076	.897	3.518	.039
α	−.859	.411		−2.090	.128

**Table 16:** ANOVA table for IMP_i_ = α + β(ASC_i_) + e_i_; R = .897; R^2^ = ; .805; Adjusted R^2^ = .740; Std Error = .135

Model	Sum of square	df	Mean square	F	p value
Regression	.224	1	.224	12.373	.039
Residual	.054	3	.018		
Total	.278	4

**Table 17:** Regression table for IMP_i_ = α + β(ASC_i_) + e_i_

Model	β (unstandardized)	Std error (of β)	β (standardized)	t	p value
ASC	2.996	.852	.897	3.518	.039
α	3.625	.503		7.211	.005

As could be seen from the above analysis and results, there exists a significant association between SI, DI, ASC and IMP either as a one tail or two tail relationship or both. There is a very high correlation between SI and ASC and between DI and ASC (r = 96 %). This shows that ASC can be used in place of either SI or DI (and vice versa) in the estimation customer satisfaction. More so, there is a high association between SI and IMP, DI and IMP; and between ASC and IMP. This implies that these Kano satisfaction scores are positively related or proportional to self-stated requirement importance (worth) and thus indicates that software engineers and designers should care about eliciting and incorporating user satisfying requirements that are of worth to users/customers in the products they are developing. Users or customers perceived importance (worth) of requirements or features have influence on the satisfaction they have if such requirements are met or if the features are incorporated into the product design. In the same vein, users/customers perceived importance of requirements or feature also have impact on the dissatisfaction they have when such requirements are not met or such features are not included in the design of the software product (i.e., from ASC = f(IMP)). The satisfaction users/customer derive when a requirement is fulfilled or a feature is placed in the product (SI or ASC) has strong influence on the value the users/customers place on such requirements/features when met (IMP). Also, the dissatisfaction users/customers received when a requirement is not met or when a feature is not incorporated into the product (as implied in ASC) also has a strong effect on the worth (IMP) of that given requirement/feature as perceived by the users or customers [however, the above effect does not exist in the case of DI = f(IMP)]. In the light of the foregoing, since satisfaction is proportional to importance, it is then necessary to give adequate attention to user/customer satisfying requirements from elicitation to design and to the final implementation of the design. Incorporating user or customer satisfying requirements in product design is of great worth or value to the future users or customers of the product.

However, the models are limited to the explanatory variable: self-stated requirements importance (IMP) which was used to explain the response variable: Kano model customer satisfaction variables (SI, DI, or ASC). Furthermore, SI, DI, and ASC were also used as explanatory variables to explain IMP (this was to provide a fuller picture of the behavior of the variables used in the models). IMP accounts for 78 % of the variability in SI; IMP accounts for 54 % of the variability in DI; and IMP accounts for 74 % of the variability in ASC. These statistics indicate the limitation of the models as it shows that there are other factors (as evidenced from the adjusted R^2^ statistics) not included in the models that account for the variability in customer satisfaction. However, this study is restricted to self-stated requirement importance and how it influences the Kano model’s customer satisfaction variables. This limits the inclusion of other explanatory variables.

## Conclusion

To summarize, a close look at the relationship between users and customers derived satisfaction (from fulfilled requirements) and the associated importance they place on such requirements or feature is quite revealing. The fulfillment or non-fulfillment of users or customers requirements affects the value they place on the given requirements. On the other hand as well, the worth of met requirement or feature also influences the level of user or customer satisfaction. This underscores the necessity of giving priority to both eliciting requirements that delight and satisfy users or customers and ensuring that those elicited requirements or features are incorporated into the design and implemented in the software product. User or customer satisfaction promotes product quality as companies not wanting to loss the patronage and loyalty of their customers will work towards satisfying them with satisfying products. This breeds a healthy competition among competing products in the market, thus, providing an enabling ground/environment for product improvement and quality, and works for a better satisfaction of the users/customers of the products. Products with features that satisfy or delight users or customers are considered of quality and perceived as important to them. So endeavoring to have those features in place in the product will add to the value and quality of the product and make the product competitive in the market.

This study is however limited due to the relative smallness of the sample size, the domain of the study (e-health awareness domain) and the coverage of participants (the participants were made up of staff and students of Universiti Utara Malaysia and they were mostly African and Asians by descent/domicile). Future confirmatory studies may leverage on these limitations by increasing the sample size, working in other domains and extending more the coverage of research participants. The sample size of 50 is a limitation to the study. However, though the sample size is relatively small, the models used in the analysis significantly fit the data except for the DI = f(IMP) model [as can be seen from the significant adjusted R^2^ and F statistics. The adjusted R^2^ for SI = f(IMP) is 78 %, for ASC = f(IMP) is 74 %, however, that of DI = f(IMP) is 54 %]. Generally speaking, this implies that the data size is fairly okay for the study.

Future works will consider further implication of the association of user or customer requirements satisfaction, the importance of such requirements and the resulting quality of the software product.
